# Social stress task with parental support or self-instruction decreases negative cognitions in children with social anxiety disorder

**DOI:** 10.1038/s41598-025-95032-8

**Published:** 2025-03-25

**Authors:** Nadine Vietmeier, Brunna Tuschen-Caffier, Julia Asbrand

**Affiliations:** 1 Department of Psychology, Faculty of Life Sciences, Humboldt-Universität zu Berlin, Unter den Linden 6, 10099 Berlin, Germany; 2 Department of Psychology, Faculty of Social and Behavioural Sciences, Friedrich Schiller University Jena, Jena, Germany; 3 Department of Psychology, Faculty of Economies and Behavioural Sciences, Albert Ludwig University of Freiburg, Freiburg, Germany

**Keywords:** Self-focused attention, Anticipatory rumination, Post-event processing, Speech task, Buffer effects, Social phobia, Human behaviour, Paediatric research, Anxiety

## Abstract

Social anxiety disorder (SAD) is highly prevalent in childhood, yet research on cognitive processes in children with SAD is limited. This study examined anticipatory rumination (AR), self-focused attention (SFA), and post-event processing (PEP) in children with SAD and healthy controls (HC), and the impact of possible influencing factors (two conditions: parental support, self-instruction). Based on adult cognitive models, we hypothesized that children with SAD would report more negative and similar positive AR, more internal SFA, similar external focus, more negative and similar positive PEP compared to HCs. We predicted that both conditions would reduce negative AR, internal SFA, and negative PEP, with stronger effects in HCs. Participants (ages 9–14; SAD: *n* = 43, HC: *n* = 48) completed two speech tasks (T1, T2) in front of a peer audience. Children with SAD reported more negative AR, internal SFA, and negative PEP than HCs in both sessions. All children reported more negative AR, internal SFA, and negative PEP in T1 than T2. Surprisingly, these effects were equally strong in both groups. Condition effects were comparable across groups, with reduced negative cognitive processes in T2. These findings highlight cognitive biases in childhood SAD and the need for further research on their modifiability.

## Introduction

With a prevalence up to 9%^1^, social anxiety disorder (SAD) is one of the most common mental disorders in childhood and adolescence. Key symptoms are an intense fear and avoidance of the scrutiny of others^2^. The typical age of onset is in late childhood to mid-teens^3^, i.e., during a time of major emotional and cognitive change. If left untreated, SAD often takes a chronic course^4^ with negative effects on various areas of life (e.g., negative peer interactions, more rejection experiences, reduced self-esteem^5^; early school drop-out^6^) well into adulthood.

Cognitive models emphasize the importance of cognitive processes in the maintenance of SAD^7^ and have been empirically well documented for adulthood^8,9^. Based on this evidence, highly effective therapies have been developed for adults with SAD which aim to modify clearly defined cognitive processes (e.g.^10,11^). In contrast, no cognitive model specific for childhood SAD currently exists^12^. Therefore, children with SAD are often treated with a generic form of cognitive behavior therapy (CBT), with considerably lower response rates than children with other anxiety disorders receiving CBT^13^. Improving treatment outcomes and efficacy in children with SAD requires a clear understanding of the cognitive processes that contribute to the maintenance of the disorder^12^.

## Relevance of cognitive models in childhood SAD

One of the most influential maintenance models of SAD so far, Clark and Wells’^7^ cognitive model, postulates that individuals with SAD interpret social situations in an excessively negative manner. These negative interpretations are maintained by different cognitive processes which can occur before, during, and after a threating situation, thus forming a vicious cognitive circle^14^.

In *anticipatory rumination* (AR^7^) prior to a social situation, individuals expect negative outcomes of the upcoming social situation based on previous alleged social failure (e.g., “Everyone will see how nervous I am. I will black out again.”). During a social situation, individuals with SAD focus excessively on their own self-related thoughts, feelings, and physical signals in a potentially threatening situation (e.g., “My hands are shaking. I need to speak more clearly”). This increased *self-focused attention* (SFA^7^) can give rise to a (distorted) image of oneself, possibly leading to negative inferences about how oneself appears to others. In *post-event processing* (PEP^7^) after a social situation, individuals review the interaction in detail, focusing on negative aspects and potentially reliving uncomfortable feelings (e.g., “This was so embarrassing!”). This may enhance a negative self-image, leading to negative expectations regarding future situations (e.g., “I always behave awkwardly”) and creating a vicious circle. While numerous studies have identified these cognitive processes as key mechanisms in the maintenance of adult SAD^8,9,15^, research in children and adolescent SAD is scarcer. In general, there are initial findings that similar cognitive factors and interconnecting processes play an important role in the maintenance of the disorder from middle childhood onwards^16,17^. However, there might be specifics such as safety behaviors or particular negative cognitions that are more prevalent in youths^18^. At this age, one developmental task is to reorganize socially, so social information becomes more relevant and processing structures need to be developed^19^. The sensitivity to social information could in turn affect the behavior in social situations^20^. At the same time, there are fewer ingrained structures compared to adults, i.e., the vicious circle is perhaps not yet established. Further research in childhood and adolescence is essential to account for both cognitive development, which progresses substantially into early adulthood, and the particular social context, which may also impact cognitive development^12^. Studies that investigate and experimentally manipulate these cognitive processes in childhood SAD can help to provide an empirical basis for child-specific models^12^.

### Empirical evidence for cognitive biases in childhood SAD

In recent years, research into the cognitive processes involved in the maintenance of SAD in childhood has become increasingly important, but studies have mostly been conducted with non- or subclinical samples (for a review see^12^). In addition, given the assumption that children with anxiety disorders differ from children without anxiety disorders in terms of negative but not positive cognitions^21^, there are hardly any studies on positive cognitive processes in children with SAD (e.g.^22,23^).

Among the few studies on AR in childhood, correlative studies indicate that high social anxiety in children is related to increased negative rumination regarding an upcoming social situation (e.g.^18,24^). In an experimental study by Lidle and Schmitz (*n* = 60, *M*_age_ = 11.6 years^23^), children with SAD reported more negative AR in preparation for a social stress task which was associated with higher subjective anxiety during the task. No differences in positive AR between children with SAD and HC children were found.

Regarding SFA, previous studies have mostly measured attention allocation using objective data (e.g., reaction time and eye-tracking^25^) while self-reports might better clarify underlying cognitive processes. Kley et al.^26^ manipulated SFA in their study with children with SAD, subclinical social anxiety and without social anxiety (*n* = 60, *M*_age_ = 10.3 years). Given the instruction to focus on oneself, all children reported more anxiety after an anxiety producing role-play compared to an external focus. In the same study, SFA mediated the relationship between general social anxiety and state anxiety in response to the task (*n* = 63, *M*_age_ = 10.3 years^27^). While a detrimental influence of SFA on social anxiety can be assumed^27^, a bidirectional relationship has been reported several times in studies from adulthood^8^. Therefore, more studies with clinical samples in childhood are necessary to better understand the role of SFA in the maintenance of childhood SAD and to assess possibly influential factors on both SFA and social anxiety.

PEP has also been studied in only a few studies with clinical samples in children. In a questionnaire study with a subclinical sample, PEP was predictive for social anxiety (*n* = 171, *M*_age_ = 12.2 years^18^). This finding is supported by an experimental study where children with social anxiety reported more negative PEP than children without social anxiety 2.5 h and one week after a social stress task (*n* = 40, *M*_age_ = 11.1 years^28^). Regarding clinical samples with SAD, Schmitz et al.^29^ found more negative and less positive PEP immediately and 2.5 h after a social stress task in children with SAD (*n* = 24, *M*_age_ = 10.2 years) compared to children without SAD (*n* = 22, *M*_age_ = 9.8 years). In the above mentioned study by Lidle and Schmitz^23^, children with SAD reported more negative PEP than children without SAD. However, there were no differences with regard to positive PEP. A brief cognitive distraction intervention did have an influence on subjective anxiety and autonomic arousal, but neither on negative nor positive PEP.

Taken together, further studies on these processes in children with SAD are needed to test the robustness of the few existing findings with clinical samples. Especially experimental studies on positive cognitions would be highly relevant, since a focus on positive thoughts is already implemented in CBT as a cognitive intervention or in preparation for exposure exercises^30,31^. For example, Asbrand et al.^22^ showed in a clinical sample (*n* = 71, *M*_age_ = 11.3 years) that positive PEP could be built up through 12 group sessions of CBT compared to a waiting group, while negative PEP showed no change. These results underline the need for further studies not only on the frequency and modifiability of negative, but also positive cognitive processes in childhood SAD.

### Influencing factors on SAD symptoms in children

Clark and Wells’ model^7^ describes causal processes that explain the increase of social anxiety in a social situation. Changes in situational factors should therefore lead to a change in cognitive distortions. The manipulation of these situational factors and causal relationships would allow an approximation of internal, automatic, and therefore difficult to investigate processes and provide an opportunity to test the model for childhood and adolescence. However, regarding childhood, it is mostly unclear which factors could possibly influence cognitive processes and reduce SAD symptoms in social situations.

In a population-based Norwegian study with 7895 adolescents (*M*_age_ = 15.9 years^32^), perceived social support and social self-efficacy were associated with lower trait SAD symptoms. Social support additionally attenuated the association between negative life events and SAD symptoms. The authors suggest interventions to modify social support and self-efficacy in at-risk adolescents. Still, research on possible underlying mechanisms unclearing concise social situations, i.e., state social anxiety symptoms, and clinical levels of social anxiety is still lacking.

Based on studies from adulthood, social support buffers moderately to strongly experienced stress, measured by cardiovascular reactions (for an overview see^33^). For example, Miers^34^ showed in a non-clinical sample with young adults (*n* = 65, *M*_age_ = 19.4 years) that positive peer-feedback after a speech lead to a higher frequency of positive PEP in high socially anxious participants compared to neutral feedback. However, studies in childhood and with clinical samples are lacking. Since sources of support seem to vary across the life span with parental support being the most relevant among children^35^, the influence of parental support on SAD symptoms in children is of particular interest.

Additionally, in the “gold standard” of treatment for anxiety disorders in children (CBT), one aim is not only to reduce anxiety but also to improve the way in which anxiety is dealt with^36^. This perceived self-efficacy is defined as the belief that one can control situational factors and events^37^. The increase in self-efficacy expectations therefore has the potential to influence negative distortions at the SFA, PEP and AR levels and rectifying the cognitive vicious circle^38^, which, to our knowledge, has not yet been investigated in children with SAD. Self-instructions have, however, been found to promote children’s self-efficacy and therefore reduce anxiety symptoms^39^ and could aim to modify interpretations positively (e.g., “I can do this!”). Based on Clark and Well’s model^7^, a more positive interpretation could modify situational anxiety and further cognitive processes (e.g., “It’s ok that my hands are shaking. I will get this nevertheless.”). Additionally, while the modification of cognitive processes is also considered a long-term process (e.g.^40^), self-instructions can be taught relatively quickly as a brief intervention and therefore be used efficiently in experiments.

### The current study

In summary, much of the research on childhood SAD is concerned with cognitive biases, but mostly relates to subclinical samples. Since SFA, AR, and PEP are key maintenance processes in adult SAD based on theoretical^15^ as well as empirical evidence^8,9^, more studies in childhood are needed to better understand cognitive processes and their modifiability in childhood SAD. Against the background of already existing treatment programs^41,42^, a better understanding of these processes would be of direct practical relevance. Therefore, we aimed to measure AR, SFA, and PEP during a well-controlled social stress task in a clinical sample of children with SAD and a healthy control (HC) group, additionally assessing possible influencing factors (parental support vs. self-instruction) prior to a repeated social stress task. We postulated the following hypotheses:

Prior to a social stress task, children with SAD will report (a) more negative AR, and (b) comparable positive AR compared to HC children^7,8,23^. During a social stress task, children with SAD will report (c) more internal SFA, and (d) a comparable external focus of attention compared to HC children^7,26^. Following a social stress task, children with SAD will report (e) more negative PEP, and (f) comparable positive PEP compared to HC children^18,23,29^ (Hypothesis 1: Group differences in AR, SFA, and PEP at T1).

A brief intervention with possible influencing factors (parental support vs. self-instruction) before a second social stress task will lead to a decrease in (a) negative AR, (b) internal SFA, and (c) negative PEP compared to a first social stress task^7,32,33^; this effect will (d) be more pronounced in HCs than in children with SAD (Hypothesis 2: Effects of repeated task with brief intervention). We analyzed the differences of this effect between both conditions as exploratory as the evidence is insufficient to date. We expect similar results for both conditions in not preregistered, exploratory analyses^32^.

## Results

### Manipulation check

Children with SAD reported higher subjective anxiety than HCs at all points, all *t*s ≥ − 8.410, all *p*s < 0.001, all *d*s ≥ − 1.766. Baseline anxiety at T1 did not differ to T2 in both groups, children with SAD: *t*(41) = 0.471, *p* = 0.320, *d* = 0.073, 95% [− 0.548, 0.882], HCs: *t*(47) = 1.286, *p* = 0.102, *d* = 0.186, 95% [− 0.100, 0.454]. Anxiety levels increased significantly from baseline to anticipation in T1 and T2 in both groups, children with SAD: *t*s ≥ − 12.394, all *p*s < 0.001, all *d*s ≥ − 1.890, HCs: *t*s ≥ − 7.853, all *p*s < 0.001, all *d*s ≥ − 1.133. Correlation coefficients between AR, SFA, and PEP are shown in Table 1.

**Table 1:** Correlation coefficients between AR, SFA, and PEP.

Variable	*M*	*SD*	1	2	3	4	5	6	7	8	9	10	11	12
1. AR positive T1	1.019	0.971	1											
2. AR negative T1	2.303	1.437	− 0.177	1										
3. SFA internal T1	1.745	0.980	− 0.099	0.709**	1									
4. SFA external T1	1.258	0.972	− 0.121	0.508**	0.583**	1								
5. PEP positive T1	1.034	1.029	0.702**	− 0.253*	− 0.107	− 0.212*	1							
6. PEP negative T1	2.013	1.528	− 0.147	0.873**	0.678**	0.515**	− 0.223*	1						
7. AR positive T2	1.244	0.992	0.619**	− 0.037	0.105	− 0.109	0.651**	0.021	1					
8. AR negative T2	1.791	1.515	− 0.138	0.790**	0.677**	0.495**	− 0.158	0.827**	− 0.003	1				
9. SFA internal T2	1.467	0.923	− 0.034	0.643**	0.851**	0.569**	− 0.083	0.592**	0.117	0.631**	1			
10. External focus T2	1.074	0.956	− 0.171	0.413**	0.508**	0.747**	− 0.169	0.466**	− 0.129	0.554**	0.567**	1		
11. PEP positive T2	1.310	1.167	0.492**	− 0.075	0.015	− 0.190	0.612**	− 0.067	0.743**	− 0.108	0.056	− 0.196	1	
12. PEP negative T2	1.616	1.560	− 0.171	0.785**	0.622**	0.567**	− 0.274*	0.835**	− 0.148	0.828**	0.636**	0.608**	− 0.233*	1

AR positive and AR negative measured by *TQ-C AR*^28,29^, range: 0–5. SFA and External focus measured by *FAQ*^26^, range: 0–4. PEP positive and PEP negative measured by *TQ-C PEP*^28,29^, range: 0–5.

*p < 0.05, **p < 0.001.

### Main analyses

The analyses revealed significant main effects for Group and Session for all dependent variables as shown in Table 2. No interaction effects between Group x Session were found (all *p*s > 0.05). Results of follow-up analyses are reported below. We provided additional analyses controlled for age in the supplements.

**Table 2:** Means, Standard Deviations of Dependent Variables and Main Effects of ANOVAs.

Dependent variable	*M* (*SD*)				*F* tests					
	SAD		HC		Group			Session		
	T1	T2	T1	T2	*F*^a^	*p*	η_*p*_^2^	*F*^a^	*p*	η_*p*_^2^
AR positive	0.717 (0.759)	1.101 (0.867)	1.288 (1.071)	1.369 (1.083)	5.268	0.024	0.056	6.688	0.011	0.071
AR negative	3.253 (1.143)	2.701 (1.430)	1.421 (1.043)	0.994 (1.084)	60.747	< 0. 001	0.408	23.279	< 0.001	0.209
SFA	2.266 (0.871)	1.885 (0.917)	1.268 (0.826)	1.102 (0.767)	27.497	< 0. 001	0.238	25.458	< 0.001	0.224
External focus	1.647 (0.931)	1.421 (1.047)	0.861 (0.771)	0.771 (0.757)	17.585	< 0. 001	0.167	4.972	0.028	0.053
PEP positive	0.739 (0.777)	1.107 (1.022)	1.253 (1.210)	1.496 (1.249)	4.150	0. 045	0.051	7.476	0.008	0.087
PEP negative	3.104 (1.295)	2.654 (1.442)	1.054 (0.984)	0.748 (1.008)	65.005	< 0. 001	0.455	14.453	< 0.001	0.156

SAD = Social anxiety disorder group. HC = Healthy control group. T1 = Session 1. T2 = Session 2. AR positive and AR negative measured by *TQ-C AR*^28,29^, range: 0–5. SFA and External focus measured by *FAQ*^26^, range: 0–4. PEP positive and PEP negative measured by *TQ-C PEP*^28,29^, range: 0–5. No interaction effects were found (all *p* > 0.05).

^a^*F*(1,88) for AR positive, AR negative, SFA and External Focus. *F*(1,78) for PEP positive and PEP negative (difference due to missing data).

#### Hypothesis 1: group differences in AR, SFA, and PEP at T1

Children with SAD reported significantly less positive AR, more negative AR, more SFA, more external focus of attention, less positive PEP, and more negative PEP than HC children at T1 (Table 3).

**Table 3:** Group differences in dependent variables at T1.

Dependent variable	*M* (*SD*)		*t* tests				
	SAD	HC	*t*	*df*	95% CI	*p*^a^	*d*
AR positive	0.718 (0.750)	1.288 (1.071)	2.910	89	0.181, 0.959	0. 005	0.611
AR negative	3.288 (1.152)	1.421 (1.043)	− 8.113	89	− 2.324, − 1.410	< 0. 001	− 1.703
SFA	2.277 (0.864)	1.268 (0.826)	− 5.693	89	− 1.361, − 0.657	< 0. 001	− 1.195
External focus	1.702 (0.988)	0.861 (0.771)	− 4.548	89	− 1.208, − 0.473	< 0. 001	− 0.955
PEP positive	0.750 (0.738)	1.276 (1.178)	2.560	80.239	0.117, 0.935	0. 012	0.526
PEP negative	3.125 (1.264)	1.063 (1.005)	− 8.565	87	− 2.540, − 1.583	< 0. 001	− 1.821

T1 = Session 1. SAD = Social anxiety disorder group. HC = Healthy control group. AR positive and AR negative measured by *TQ-C AR*^28,29^ ranged from 0 to 5. SFA and External focus measured by *FAQ*^26^ ranged from 0 to 4. PEP positive and PEP negative measured by *TQ-C PEP*^28,29^ ranged from 0 to 5.

^a^Two-tailed *t* tests for AR positive, External focus, and PEP positive; one-tailed *t* tests for AR negative, SFA, and PEP negative.

#### Hypothesis 2: effects of repeated task with brief intervention

**Fig. 1:**
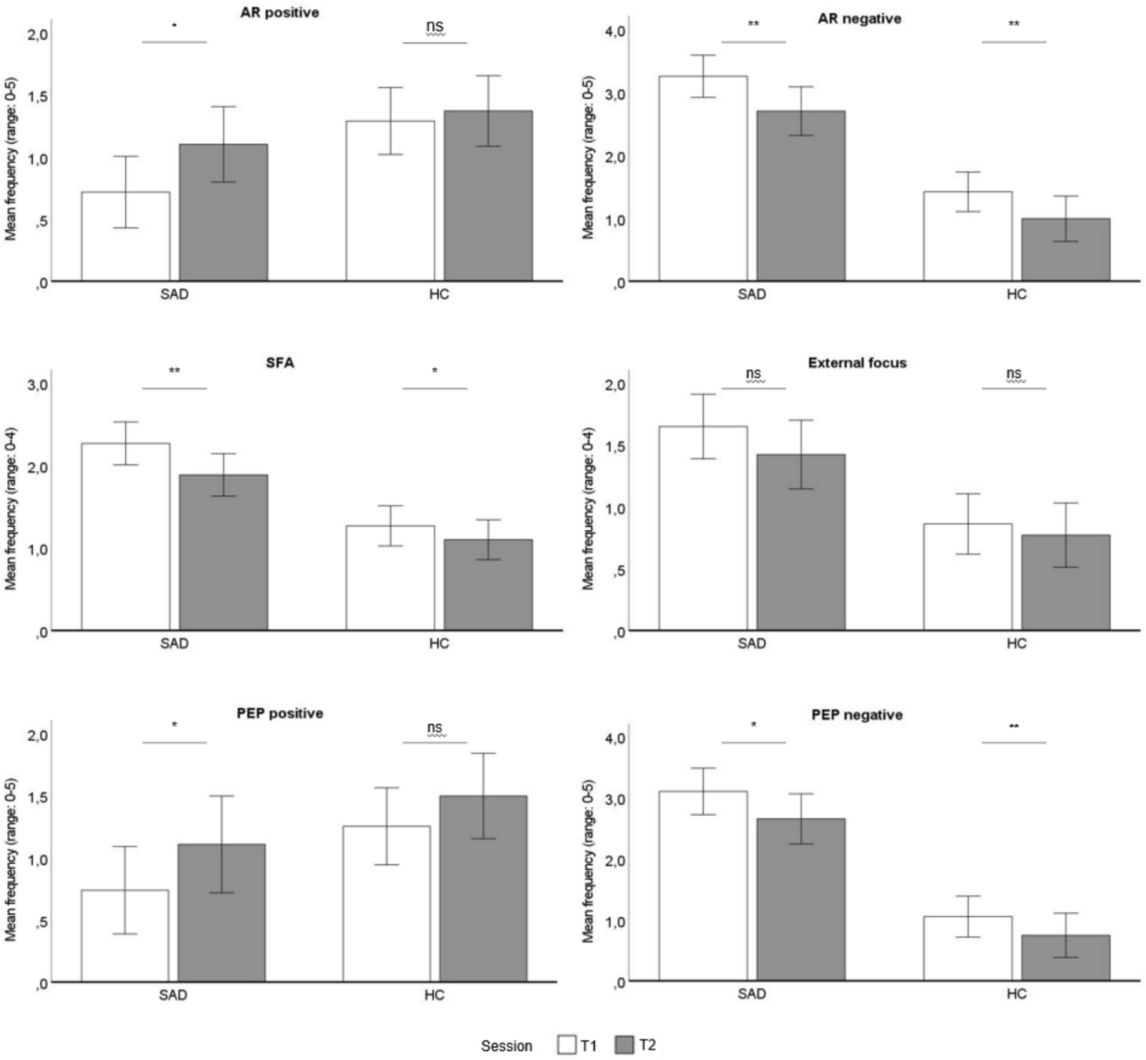
Differences of mean frequency in dependent variables between T1 and T2. *Note.* SAD = Social anxiety disorder group. HC = Healthy control group. T1 = Session 1. T2 = Session 2. AR positive and AR negative measured by *TQ-C AR*^28,29^. SFA and External focus measured by *FAQ*^26^. PEP positive and PEP negative measured by *TQ-C PEP*^28,29^. Error bars indicate 95% confidence intervals. *p < 0.05, **p ≤ 0.001, ns = not significant.

Differences in all dependent variables between T1 and T2 for both groups are shown in Fig. 1.

*AR*. Both groups reported significantly more negative AR in T1 than T2, children with SAD: *t*(41) = 3.217, *p* = 0.001, *d* = 0.496, 95% [0.205, 0.898], HCs: *t*(47) = 3.674, *p* < 0.001, *d* = 0.530, 95% [0.193, 0.661]. Children with SAD reported significantly more positive AR in T2 than in T1, *t*(41) = − 3.294, *p* = 0.002, *d* = − 0.508, 95% [− 0.619, − 0.149], while we found no difference for HCs, *t*(47) = − 0.605, *p* = 0.548, *d* = − 0.087, 95% [− 0.350, 0.188].

*Focus of attention*. Children in both groups reported significantly higher SFA in T1 than in T2, children with SAD: *t*(41) = 4.025, *p* < 0.001, *d* = 0.621, 95% [0.190, 0.572], HCs: *t*(47) = 2.836, *p* = 0.003, *d* = 0.409, 95% [0.048, 0.285]. Both groups reported a higher external focus of attention in T1 than in T2. However, the results were not significant, SAD: *t*(41) = 1.882, *p* = 0.067, *d* = 0.290, 95% [− 0.017, 0.469], HCs: *t*(47) = 1.113, *p* = 0.271, *d* = 0.161, 95% [− 0.073, 0.253].

*PEP*. Children with SAD (*t*(34) = 2.384, *p* = 0.011, *d* = 0.403, 95% [0.066,0.834]) and HCs (*t*(44) = 3.184, *p* = 0.001, *d* = 0.644, 95% [0.112, 0.499]) reported more negative PEP in T1 than in T2. Children with SAD showed significantly less positive PEP after T1 compared to T2, *t*(34) = − 3.170, *p* = 0.002, *d* = − 0.536, 95% [− 0.604, − 0.132], while we found no difference for HCs, *t*(44) = -1.390, *p* = 0.171, *d* = − 0.121, 95% [− 0.597,0.109].

### Exploratory analysis: effect of condition

Regarding negative AR (all *p*s ≥ 0.374), internal SFA (all *p*s ≥ 0.067), external focus of attention (all *p*s ≥ 0.389), negative PEP (all *p*s ≥ 0.561), and subjective anxiety (all *p*s ≥ 0.121), all main effects of Condition and interaction effects including Condition were nonsignificant.

For positive AR, exploratory analyses revealed a main effect for Condition, *F*(1,85) = 14.050, *p* = 0.002, η_*p*_^*2*^ = 0.109, with children in the condition with positive self-instruction reporting significantly more positive AR at T2, *t*(87) = -3.569, *p* < 0.001, *d* = − 0.757, 95% [− 1.102, − 0.314]. However, this effect was already present at T1, *t*(87) =  − 2.038, *p* = 0.045, *d* = − 0.432, 95% [− 0.818, − 0.010]. All interaction effects including Condition were nonsignificant (all *p*s ≥ 0.110).

For positive PEP, exploratory analyses revealed a main effect for Condition, *F*(1,75) = 11.489, *p* = 0.001, η_*p*_^*2*^ = 0.133, with children in the condition with positive self-instruction reporting significantly more positive PEP at T2, *t*(73.417) = − 3.599, *p* < 0.001, *d* = − 0.783, 95% [− 1.327, − 0.381]. Like with positive AR, this effect was already present at T1, *t*(76.159) = -2.609, *p* = 0.011, *d* = − 0.551, 95% [− 0.969, − 0.130]. All interaction effects including Condition were nonsignificant (all *p*s ≥ 0.145).

## Discussion

Our aim was to examine differences in cognitive processes before (AR), during (SFA), and following (PEP) a social stress task in front of same-aged peers between children with SAD and HC children. Additionally, we tested the effects of non-standardized parental support versus a self-chosen positive self-instruction given before a repeated social stress task on the assessed cognitive processes.

As expected, children with SAD reported more negative AR, more internal SFA, and more negative PEP compared to HC children. These results are in line with the few previous experimental studies with clinical samples, e.g., examining PEP^28,29^, both AR and PEP^23^, AR and PEP regarding social performance^43^, and SFA^26,27^, suggesting the relevance for Clark and Wells’ cognitive model^7^ also for childhood SAD.

However, only partly confirming our hypothesis, children with SAD did not report an expected comparable, but higher external focus of attention than HC children during the task. In a previous study, Kley et al.^27^ also used the same questionnaire, FAQ^27^, for attention assessment in children and found no effects between a clinical SAD, a subclinical and nonclinical group. Their experimental manipulation of attention allocation limits the comparability to our study, though. As these and our results are based on subjective self-reports, they do not necessarily reflect actual (behavioral) attentional processes but may better capture individual beliefs about attention allocation^44^. Studies in children with SAD using reaction time paradigms^45^ or eye-tracking^46–48^ for the assessment of attention allocation underline an attention to threat, which is also discussed as a possible maintenance factor in adults with SAD (e.g.^49^). However, if attention is more directed both internally and externally in children with SAD—as in our study—this proposes a general hypervigilance^50^ where instead of a change from internal to external attention, a *reduction* in attention allocation (e.g., relaxation techniques) may be the corresponding goal. Additionally, based on Clark and Well’s cognitive model^7^, not only a *change* from external to internal attention, but a modified *interpretation* of attentional processes could modify situational anxiety and further cognitive processes (e.g., “It’s ok that my hands are shaking. I will get this nevertheless.”). Studies that measure the interplay of attentional and cognitive processes using subjective and objective data (e.g., reaction time, eye tracking^25,51^) simultaneously are needed to better understand attention allocation in childhood SAD.

Partly contrary to our hypotheses, children with SAD reported less positive AR and PEP than HCs in T1. Previous studies reported no difference in positive PEP^22^, positive AR and PEP^23^ or less positive PEP^29^ in children with SAD compared to HC children. Our study cannot confirm the possible age difference discussed as a possible reason for the inconsistent results (cf.^22^), even showing a medium^52^ effect (*d* = 0.611) in a sample with a mean age of 12.1 years. However, the frequency of both processes was, as expected, comparable for both groups in T2, proposing that positive AR and PEP are not only relevant, but also efficiently modifiable outcome variables in childhood SAD. Since negative and positive thoughts in children were shown to be relatively independent in children in general^53^, future studies should aim to investigate further possible influences on positive cognitions.

Supporting our hypotheses, children in both groups reported less negative AR, internal SFA, and negative PEP in T2 compared to T1. Surprisingly, and contrary to our hypotheses, this effect was similarly pronounced in children with SAD and HCs: We found no interaction effects between Group x Session for all assessed variables, suggesting that children with SAD benefited equally from time, repetition, and/or buffering conditions like HCs concerning cognitive variables. So far, only a few studies have conducted repeated social stress tasks in children with SAD, with short to long time periods between tasks, thus limiting comparability. Potentially, the one-week time period between T1 and T2 led to a change of preparation of the children with SAD (e.g., more preparation in terms of safety behaviour^7^), and thus to a reduction in negative cognitions. At the same time, however, more preparation time could also lead to increased AR (cf.^54^). Future studies may monitor the extent to which children have prepared for the second task in the meantime (e.g., via self-report questionnaires) to assess potential influences. Additionally, psychophysiological assessments could provide further insight into stress reactivity^55^ of repeated exposure and possible influences on cognitive variables in children with SAD in future studies.

The type of buffering condition showed a significant effect for positive AR and PEP, which was already present at T1 and therefore cannot be attributed to the conditions given in the anticipation phase of T2. First, it is possible that the sample differed randomly from T1. However, we found no differences in age and gender, and children with SAD and HCs were equally distributed across the conditions. Second, although the participants were blinded to the condition at T1, the experimenters already knew at T1 which condition the child would receive due to organizational processes. Therefore we cannot rule out possible experimenter effects^56^ which may have led to the differences in positive AR and PEP at T1 for children in the self-instruction condition. Further blinded studies are needed to avoid possible biases and test the robustness of our results.

Extending previous studies, we examined AR, SFA, and PEP simultaneously. Our preliminary analyses revealed significant, mostly strong correlations between negative AR, SFA, external focus, negative PEP at T1 and T2 (*r* = 0.413–0.873). Similar to SAD in adulthood (e.g.^7,57^), this suggests a vicious circle, in which the interplay of the individual negative cognitive processes may maintain social anxiety. Supporting the combined cognitive biases hypothesis^58^ which states that cognitive biases do not operate in isolation but are one part of a set of processes, we suggest that it may be more useful for future studies to investigate how biased cognitive processes affect each other and how they can be positively influenced, i.e., how the vicious circle can be broken. This includes longitudinal studies to better understand the role of cognitive processes in the course and maintenance of SAD.

There are several limitations in our study that should be considered for future research. First, the lack of a control condition considerably limits the interpretation of the buffering effects. While we had initially planned to conduct a third session so that all children underwent both conditions in randomized order, the study had to be adapted due to the COVID-19 and accompanying restrictions, thus leading to the necessity to limit laboratory sessions. Since we could not find any differences between the two conditions about the variables surveyed, it is therefore not possible to conclude whether the effects at T2 are due to a time effect, a repetition of the task (e.g., mere exposure^59^), or the buffering conditions. The exploratory findings regarding the buffer effects should therefore be interpreted with caution. Nevertheless, we found significant and comparable effects from T1 to T2 in both groups, providing valuable approaches for future research. To further test the effect of both conditions, we suggest that future studies should include a no-intervention control group.

Second, while all children were told that the audience was connected live, a few children expressed their doubts during the debriefing that they might had seen a video recording. Although Westenberg et al.^54^ showed that a fear-induction is successful even if the participants are told beforehand that the audience is pre-recorded, we cannot rule out the possibility that different degrees of fear induction may have occurred in our study. However, initial analyses in our study showed successful anxiety induction in both groups. A systematic survey of the subjects’ subjective impression of the authenticity of the audience may be controlled for as a possible influencing factor in future studies.

Third, we did not provide instructions on how the content of the brief interventions in T2 should be structured (e.g., specific supportive behavior, providing the same self-instruction for each child). While we wanted to achieve high ecological validity, it can be assumed that the condition effects for children with SAD differs from that of HC children. For example, previous studies suggest that parenting behaviour may differ between parents of children with SAD and HC children^60^ and the effect of self-instructions depend on, e.g., task complexity^61^, which is presumably higher for children with SAD than HC children. Additionally, in line with preregistration procedures, children in both buffering conditions were asked to find a helpful self-instruction at the end of T1; therefore, we cannot exclude the possibility that children in the parental support condition also remembered and used their self-instruction at T2.

Fourth, we aimed to maximize ecological variability while keeping a largely standardized process by, for example, using unstandardized instructions for both buffering conditions which are closely related to potential strategies in real life (i.e., seeking parental support or using positive self-instructions) and a peer video audience instead of a live adult audience. However, a real performance or social situation can only be replicated to a certain extent in the laboratory. Therefore, ecological validity needs further research by decreasing standardization but using real life scenarios (e.g.^62,63^). High standardization of laboratory studies enhances internal validity^64^, which was particularly important for our study. This allowed us to minimize potential external influences while conducting exploratory analyses of the conditions possibly affecting cognitive processes. In view of the relevance of the context in which a social situation takes place, it would therefore be necessary to assess cognitive biases under everyday conditions in addition to investigating them in the laboratory^62^. Nevertheless, our study is one of the first to examine multiple cognitive processes in a repeated stress task in a clinical sample, providing a starting point for further research to better understand the cognitive processes relevant in the maintenance of this highly prevalent disorder.

In summary, our results underline the findings of the few experimental studies with clinical samples and provide empirical support for the relevance of cognitive processes in childhood SAD. Children with SAD reported more negative AR, SFA, and negative PEP than HC children, while both groups reported a decrease in these variables in a repeated speech task with parental support or self-instruction. Further research is warranted to better understand these situational determinants, including the use of psychophysiological measures of social anxiety and contexts outside the laboratory. From a clinical perspective, based on this, disorder-specific interventions should be developed to improve the still low treatment response for children with SAD.

## Methods

### Ethical approvals and consent procedures

The study was approved by the ethics committee of the University of Freiburg, Germany (application no. 24/19) and performed in accordance with the ethical standards of the institutional research committee and the 1964 Declaration of Helsinki and its later amendments or comparable ethical standards. Informed consent from all the study participants and their legal guardians was obtained after written and verbal information. Children in the clinical group were offered treatment in the department’s outpatient clinic or given further mental health care contacts.

### Trial design

The study was registered in the German Clinical Trials Register (DRKS00018880) and with the German Research Foundation (AS 553/1-1) prior to recruitment. It employed a 2 × 2 (× 2) mixed repeated measures design with one between factor (Group: SAD, HC) and one within factor (Session: T1: baseline without buffering condition, T 2: buffering condition), and, for exploratory analysis, a second between factor (Condition at T2: parental support, self-instruction). Participants were allocated by drawing from a hat to one of the conditions (block randomization). The preregistered plan, in which all participants completed both conditions in randomized order, had to be adjusted due to the Covid pandemic. During the pandemic, strict health regulations had to be observed (e.g., social distancing rules, hygiene measures) and resources (e.g., laboratory rooms, staff) were considerably limited due to lockdowns and quarantine measures. This led to a reduction in the number of possible sessions, which meant that not all the originally planned sessions could be held. The further procedure was carried out as preregistered. The variables included in this study were used as primary outcome variables for a comparison between children with and without SAD. Given the length limitations, additional analyses will be reported elsewhere. A power analysis for the present study resulted in a necessary sample size of *n* = 52 with an expected effect^15,19,21,22^ in the range of *f* = 0.40 (α = 0.05; β = 0.80) for the main effects with a two-group design (ANOVA). As the current study was part of a larger research project that required a sample size of *N* = 92, all children were included to increase power.

### Recruitment and participants

Children between 9 and 14 years were recruited by postal mail via population register data, schools, social media, and daily newspapers. Interested families (*n* = 224) were asked about SAD and other psychopathological symptoms in a telephone screening. If the screening assessment suggested eligibility, families (*n* = 138) were invited to diagnostic interviews (Kinder-DIPS^65,66^) and asked to complete online questionnaires on sociodemographic data, anxiety symptoms, and general psychopathology. Based on this child and parent information combined with clinical impression, diagnoses according to the Diagnostic and statistical manual of mental disorders (DSM-5^67^) were assigned under the supervision of a licensed child and adolescent psychotherapist and a licensed psychotherapist with additional qualification for children and adolescents. Children with a suspected or previous diagnosis of autism spectrum disorder, acute suicidal tendencies, or an overall cognitive ability below an IQ = 80 (assessed via school affiliation), children taking medication that could potentially influence fear responses or alter psychophysiological parameters (e.g., anxiolytics), and siblings were excluded. Ten participants with SAD were excluded, after T1 had to be discontinued due to excessive stress on the child. There was no difference in social anxiety scores between the dropouts and the further participants (*p*s > 0.05).

The final sample consisted of 43 children with a primary diagnosis of SAD (and possibly other mental illnesses) and 48 HC children without a lifetime diagnosis of mental illnesses. Participant characteristics are shown in Table 4. All participants spoke German fluently. The following comorbid disorders were present in the group with SAD: depressive disorders (*n* = 9), specific phobia (*n* = 7), child separation anxiety disorder (*n* = 2), insomnia (*n* = 2), generalized anxiety disorder (*n* = 1), agoraphobia (*n* = 1), posttraumatic stress disorder (*n* = 1), tic disorder (*n* = 1), enuresis (*n* = 1), and attention deficit hyperactivity disorder (*n* = 1). No participant had ever received psychotherapy or pharmacotherapy for SAD. One child took regularly medicine (asthma spray, *n* = 1).

**Table 4:** Participant characteristics.

Characteristic	Group		Statistics
	SAD	HC	
Sample size (*n*)	43	48	
Mean age (*SD*), in years	11.9 (1.5)	12.3 (1.5)	*ns*^b^
Female (%)	67.4	50.0	*ns*^c^
Mean SDQ (*SD*)	15.7 (5.3)	8.53 (5.3)	*p* < 0.001^b^
Mean SDQ—mother (*SD*)	12.2 (5.3)	4.7 (4.0)	*p* < 0.001^b^
Mean SDQ—father (*SD*)	11.4 (5.6)	5.8 (4.6)	*p* < 0.001^b^
Mean SPAI-C (*SD*)	29.9 (9.9)	7.7 (6.6)	*p* < 0.001^b^
Mean SASC-R-D (*SD*)	29.0 (6.9)	16.8 (4.8)	*p* < 0.001^b^
Mean SASC-R-D—mother (*SD*)	60.5 (11.1)	31.8 (9.9)	*p* < 0.001^b^
Mean SASC-R-D—father (*SD*)	52.6 (10.5)	35.0 (10.8)	*p* < 0.001^b^
Mean DIKJ (*SD*)	22.2 (10.4)	8.5 (5.7)	*p* < 0.001^b^
School^a^			
Primary school (%)	27.9	37.5	*ns*^c^
Grammar school (%)	11.6	12.5	*ns*^c^
Comprehensive school (%)	48.8	41.7	*ns*^c^
Other (%)	6.6	4.2	*ns*^c^
Not specified (%)	0.0	2.1	*ns*^c^
Child lives primarily with…^a^			
Both parents (%)	76.7	83.3	*ns*^c^
Mother (%)	14.0	10.4	*ns*^c^
Mother and female partner (%)	2.3	0.0	*ns*^c^
Mother and male partner (%)	7.0	2.1	*ns*^c^
Father and male partner (%)	0.0	2.1	*ns*^c^

SDQ = Strength and difficulties questionnaire—German version^68^, range: 0–40. SPAI-C = social phobia and anxiety inventory for children—German version^69^, range: 0–42. SASC-R-D = social anxiety scale for children-revised—German version^70^, range: 18–90. DIKJ = Children’s Depression Inventory^71^, range: 0–56.

^a^Based on maternal report.

^b^Based on *t* test.

^c^Based on *chi-square* test.

## Materials

### Diagnostic assessment

#### Diagnostic interview for mental disorders in children and adolescents (Kinder-DIPS)

The Kinder-DIPS^65,66^ is a structured diagnostic interview that facilitates a standardized clinical assessment of lifetime diagnoses, including current and past conditions, based on the criteria established in the 10th revision of the International Statistical Classification of Diseases and Related Health Problems (ICD-10^72^) and the DSM-5^67^. The interview was administered separately to both the child and a parent. All interviewers received comprehensive training in administering the interviews. The interviews were conducted online to minimize face-to-face contact during the Covid pandemic and videotaped for ongoing supervision by a licensed psychotherapist. The Kinder-DIPS shows high to very high interrater reliability for lifetime diagnoses (child report: κ = 0.90–0.98, parent report: κ = 0.88–0.97^73^), good test–retest reliability (child report: 75–98%, parent report: 89–100% after one week^74^), and successful validation with disorder-specific questionnaires^66^. Parents and children agree moderately on psychopathological symptoms^75^.

#### Strength and difficulties questionnaire (SDQ)

General psychopathology was assessed using the child and parent report German versions of the SDQ^68^. The SDQ is a 25-item behavioral screening with the subscales emotional symptoms, conduct problems, hyperactivity-inattention, peer relationship, and prosocial behavior. Each subscale consists of five items and is rated on a 3-point scale (“not applicable”, “partially applicable”, “clearly applicable”). While the internal consistency of the overall scale was satisfying, the five subscales only showed low reliability (α = 0.55–0.77; *r*_*tt*_ = 0.58–0.67^76^). The internal consistency in the current sample showed similar results (child report: α = 0.577–0.811, mother report: α = 0.558–0.864, father report: α = 0.425–0.836).

#### Social phobia and anxiety inventory for children (SPAI-C)

The German version of the SPAI-C^69^ evaluates behavioral traits associated with SAD. The self-report questionnaire comprises 26 items (e.g., “I feel anxious when meeting new boys or girls”), including nine sub-items that differentiate anxiety levels based on audience type (i.e., familiar and unfamiliar boys and girls, adults). Participants respond on a 3-point Likert scale, ranging from “never or hardly ever” to “almost always or always.” In a German sample^69^, the SPAI-C demonstrated excellent internal consistency and test–retest reliability over a 4 weeks period (Cronbach’s α = 0.92; *r*_*tt*_ = 0.84). The current sample exhibited excellent internal consistency (α = 0.982).

#### Social anxiety scale for children-revised (SASC-R-D)

The child and parent report German versions of the SASC-R-D^70^ consists of 22 items each, designed to assess social anxiety symptoms in children aged 8 to 16 years. The items are organized into two subscales: *fear of negative evaluation* (e.g., “I worry about what other kids think of me”) and *social anxiety and distress* (e.g., “I feel nervous when I talk to kids I don’t know very well”). Responses are rated on a 5-point Likert scale ranging from “not at all” to “all the time”. The authors of the German version reported acceptable test–retest reliability (*r*_*tt*_ = 0.67) and internal consistency (α = 0.67). The internal consistencies in the current sample were excellent (child report: α = 0.950, mother report: α = 0.933, father report: α = 0.966).

#### Children’s depression inventory (CDI)

Depressive symptoms were assessed using the CDI^71^. Children rate the severity of 26 depressive symptoms on a 3-point Likert scale from “not existing” to “strong expression”. Scores have been shown to significantly correlate with clinicians’ assessments and with objective behavioral measures of depression. Internal consistency coefficients range from α = 0.87 (school sample) to α = 0.92 (clinical sample). In the current sample, the internal consistency was excellent (α = 0.940).

#### Psychometric measurements at T1 and T2

*Subjective Anxiety* Children reported their subjective anxiety level using a child-friendly VAS^28,29^ ranging from 0 (“no anxiety”) to 10 (“extreme anxiety”).

*Focus of Attention Questionnaire (FAQ)* A modified German version of the self-report FAQ^26^ was used to assess two directions of attention regarding a social situation: internal SFA (Self-Focus Scale, 8 items; e.g., “I paid attention to what was happening in my body”) and external attention to the environment or an interaction partner (Other-Focus Scale, 3 items; e.g., “I paid attention to how the other people felt”). Children indicate on a 5-point Likert scale from “not at all” to “the whole time” to what extent they focused on certain aspects of the social situation. In a previous German study, the internal consistency was acceptable (FAQ_self_: α = 0.78, FAQ_extern_: α = 0.58^26^). In the current sample, the internal consistencies were excellent for the Self-Focus Scale (T1/2: α = 0.904/0.908) and low for the Other-Focus Scale (T1/2: α = 0.593/0.648).

#### Thoughts questionnaire for children (TQ-C)

The TQ-C^28,29^ is a 14-item self-report questionnaire designed to measure the frequency of positive and negative PEP following a social stress task (“How often did you think…”; *TQ-C PEP*). Items include cognitions regarding the performance on the social stress task (e.g., “My presentation was exciting” [positive]), perceptions of observers (e.g., “The observers found my performance poor” [negative]), and the emotions experienced (e.g., “I felt anxious” [negative]). Participants rate the frequency of each cognition using a 6-point Likert scale from “never” to “very often”. To assess AR, a parallel version was created by rephrasing the items (*TQ-C AR*;^23,77^) to focus on the upcoming social stress task (e.g., “I am going to do well on the task”). In the current sample, the scales for negative (T1/2: α = 0.944/0.961) and positive AR (T1/2: α = 0.911/0.922) exhibited excellent internal consistency. For PEP, the internal consistency was excellent for the negative scale (T1/2: α = 0.949/0.963), and high to excellent for the positive scale (T1/2: α = 0.866/0.946).

### Social stress task

A 5 min informal speech task was adapted from Westenberg et al.^54^ and Miers^34^. Participants were asked to present a topic of choice in front of a peer audience. Examples for possible topics were offered (e.g., hobbies, vacation, personal life) but not specified to minimize between-subject differences in familiarity or knowledge concerning the topic (cf.^34^). The participants were instructed that their presentation should be “as interesting as possible so that the audience is interested in getting to know [them] better”. To maintain the social-evaluative nature of the situation, the participants were informed that the presentation would be videotaped, and that the audience would be watching them via an online Zoom connection. If a child showed difficulty in beginning their speech or paused for more than 20 s speaking, the experimenter encouraged the child appropriately^54^.

The pre-recorded, neutral-looking audience consisted of six children in the same age range as the participants. We decided on a pre-recorded audience (instead of, e.g., protocols with live confederates^78^) to create a controllable and comparable setting across all participants with similar-aged peers. Different recordings of the audience (e.g., different clothing and order) were shown in T1 and T2 to improve the manipulation.

### Experimental conditions

For T2, participants received either (1) parental support or (2) a helpful self-instruction during the anticipation phase. In the parental support condition, the parent who accompanied the child to the appointment was asked to join the child at the beginning of the anticipation phase. After the parent sat down next to the child, the experimenter gave the instruction to prepare the following speech together: “This time, your mother / father is allowed to help you to prepare the speech.” No further instructions were given on how the parent should support the child to aim for maximal ecological validity. After the anticipation phase, the parent was asked to leave the room again.

A self-instruction was formulated with each child at the end of T1 to keep the T1 procedure comparable for all participants. Therefore, the experimenter asked the participant: “Sometimes certain thoughts can be helpful when we are in stressful situations, e.g., when we have to give a presentation. Do you have an idea of a thought that could have helped you today? This could be a sentence that you could tell yourself beforehand to make the presentation easier, or a motto that could give you courage”. If a child could not think of a helpful instruction themselves, the experimenter helped them to develop one by asking specific questions (e.g., “What would you say to a good friend?”, “Do you remember a sentence that helped you in the past?”). If a child reported more than one sentence, it was asked to select the most helpful one. In T2, all children in the self-instruction condition saw the selected self-instruction on an index card (DIN A6 format) in front of them on the table. They were asked to repeat the self-instruction out loud or in their heads throughout the preparation phase.

### Procedure

**Fig. 2:**
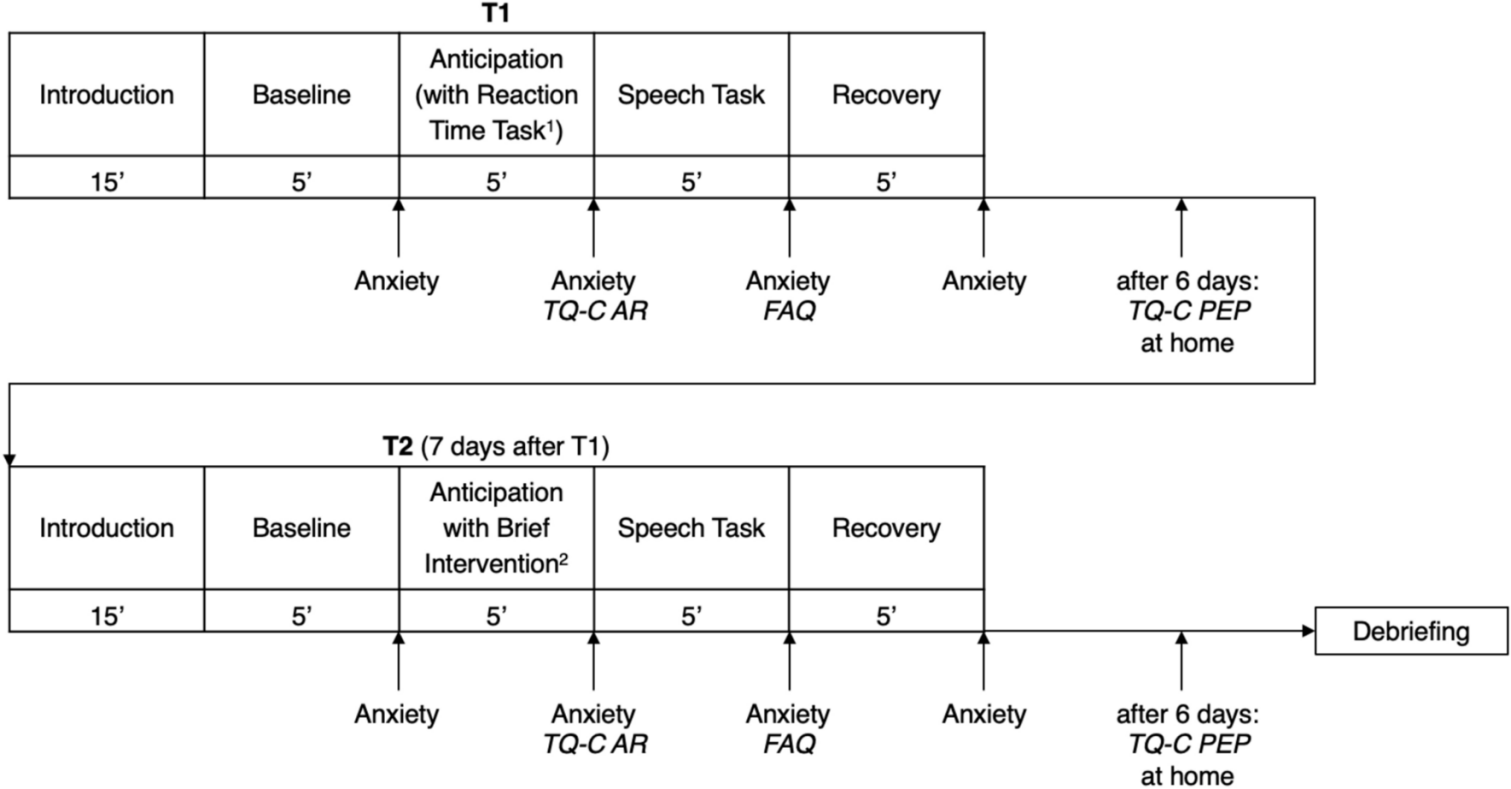
Procedure of laboratory sessions. *Note. Anxiety* = Children’s retrospect anxiety rating on a VAS scale (0–10). *TQ-C AR* = Thought’s Questionnaire for Children—Anticipatory Rumination^28,29^. *FAQ* = Focus of Attention Questionnaire^26^. *TQ-C PEP* = Thought’s Questionnaire for Children – Post-Event Processing^28,29^. ^1^The *Reaction Time Task* was not relevant for this study. ^2^Participants were randomized to receive one of two brief interventions during the anticipation phase: natural (uninstructed) parental support vs. self-chosen self-instruction.

If a child met the inclusion criteria, they were invited for two laboratory sessions (Fig. 2). Electrocardiogram (ECG), electrodermal activity (EDA), and mobile eye tracking were recorded throughout, but could not be evaluated due to technical difficulties (ECG, EDA) or will be focus of future publication (eye tracking).

Both sessions began with a 15 min warm-up phase during which the child played a board game with the experimenter. During a baseline period, participants watched a slideshow of landscape pictures, sitting at a small table in front of a computer screen. For the following anticipation phase, the child was instructed to prepare for a presentation “only in their heads”. After two minutes of preparation, the experimenter turned on the computer monitor showing the audience. After the following speech task, the child had a 5-min recovery period. At the end of T1, the experimenter asked the child to provide a helpful self-instruction. Six days after the appointment, the child was asked to complete the *TQ-C PEP* at home and bring it to T2.

T2 followed exactly one week after T1 (cf.^34^). Besides of a reaction time experiment, which was not relevant for the current study, and the brief intervention during the anticipation phase in T1, T2 followed the same procedure as T1. For the speech, children should choose a different topic than the first time.

After the second *TQ-C PEP* had been returned and thus the study participation successfully completed, the participants were thanked and debriefed. As compensation, children received a voucher for €60 and parents a bank transfer of €40.

#### Analysis/statistics

Statistical analyses were conducted using IBM SPSS Statistics for Windows, Version 29.0.2.0. The level of significance was set at α = 0.05 for all statistical analysis. Children were excluded from an analysis if ≥ 50% of responses were missing in the corresponding questionnaire.

For all hypotheses, repeated measure ANOVAs with Group (SAD, HC) as the between-subject factor and Session (T1, T2) as the within-subject factor were conducted. For the exploratory analyses, Condition (parental support, self-instruction) was listed as a second between-subjects factor. Since ANOVAs with repeated measures are relatively robust against violations of the normal distribution assumption^79^, no corresponding corrections were made in case of a violation of normal distribution. For follow-up analyses, independent *t* tests were conducted to examine differences between groups and tasks and one-sample *t* tests for differences between sessions. According to our hypotheses, the *t* tests for anxiety levels, negative PEP, SFA, and negative PEP were one-tailed and two-tailed for positive AR, external focus, and positive PEP. Degrees of freedom were adjusted according to Greenhouse–Geisser, respectively.

## Supplementary Information

Supplementary Information.

## Data Availability

Data cannot be shared publicly as this is not included in the informed consent by participants and the mental health data is particularly sensitive. However, deidentified participant data with annotations will be made available to other researchers upon reasonable request.
