# Supplementary material for: Social stress task with parental support or self-instruction decreases negative cognitions in children with social anxiety disorder
Source: Sci Rep. 2025 Mar 25;15:10220. doi: 10.1038/s41598-025-95032-8 (PMC11937552; doi:10.1038/s41598-025-95032-8)
Supplement: Supplementary file 1 — Supplementary Information. [file 41598_2025_95032_MOESM1_ESM.pdf]

SUPPLEMENTARY INFORMATION

Social Stress Task With Parental Support or Self-Instruction Decreases Negative Cognitions in Children With Social Anxiety Disorder

Nadine Vietmeier[1,2*], Brunna Tuschen-Caffier[3], Julia Asbrand[1,2]

[1]Department of Psychology, Humboldt University of Berlin, Germany

[2]Department of Psychology, Friedrich Schiller University Jena, Germany

[3]Department of Psychology, Albert Ludwig University of Freiburg, Germany

Supplementary Information

Results With Age as a Covariate

To account for possible age effects, we provide additional results for the main effects and interaction effects regarding the cognitive processes (AR positive, AR negative, SFA, external focus, PEP positive, and PEP negative), controlled for age in months. It should be noted that the influence of age was not a primary research question in this study. The present results are therefore exploratory and should be interpreted cautiously due to the post-hoc nature of the analyses.

Analysis / Statistics

Statistical analyses were conducted using IBM SPSS Statistics for Windows, Version 29.0.2.0. The level of significance was set at $\alpha = .05$ for all statistical analysis. Children were excluded from an analysis if ≥ 50% of responses were missing in the corresponding questionnaire.

For additional analyses, mixed ANOVAs with Group (SAD, HC) as the between-subject factor and Session (T1, T2) as the within-subject factors and age (in months) as a covariate were conducted. The Greenhouse-Geisser adjustment was used to correct for violations of sphericity. Since ANOVAs with repeated measures are relatively robust against violations of the normal distribution assumption[1], no corresponding corrections were made in case of a violation of normal distribution. Due to multiple testing, the alpha level was adjusted according to Bonferroni-Holm[2].

A post-hoc power analysis was conducted using G*Power[3], assuming a small effect size (f = 0.25) and a type 1-error rate of $\alpha = .05$ for an ANCOVA with fixed effects, main effects, and interactions. Considering the given sample of N = 91 with 4 groups (2x2 measurements) and df = 1, the power analysis revealed a power of 65.48%.

Main Analyses

As shown in Supplementary Table S1, the analyses revealed significant main effects for Group for all dependent variables except AR positive and PEP positive. Main effects for Session and Age were

not significant (both *p*s > .05). We found no interaction effects for Group x Session and Session x Age (all *p*s > .05; see Supplementary Tab. 2). For post-hoc analyses refer to the main manuscript.

Brief Discussion

Taking age into account, we still found differences between children with SAD and children without SAD regarding negative cognitive processes (AR negative and PEP negative) and the reported allocation of attention (SFA, external focus). In contrast to the results in which age was not considered, the analyses with age as a covariate no longer showed any difference regarding positive cognitive processes (AR positive and PEP negative). Additionally, we no longer found a main effect of Session, which was significant for all cognitive processes examined when not taking age into account.

However, given the post-hoc nature of the analyses and the low statistical power, the results should be interpreted with caution. We planned and preregistered a study procedure with the aim to compare children with SAD and children without SAD at a similar age when the disorder first emerges[4].

Based on the results in the supplements, a difference between the groups would further emphasize the relevance of negative cognitive processes and reported attention allocation in children with SAD. However, the assumption that the cognitive processes can be changed by a second stress task with parental support or self-instruction, contrary to the model of Clark and Wells[5], could no longer be supported. Furthermore, we could not confirm the relevance of positive AR and positive PEP, which would be in line with the theoretical assumption that children with anxiety disorders show no difference in positive cognitions in comparison to children without anxiety disorders[6]. As age may have a relevant influence on whether changes in cognitive processes occur, further studies on cognitive processes over the course of development in children with SAD are necessary.

Supplementary Table S1

Main Effects of ANOVAs with Age as a Covariate

Dependent variable	*M* (*SD*)				*F* tests								
	SAD		HC		Group			Session			Age		
	T1	T2	T1	T2	F^a	p	η_p^2	F^a	p	η_p^2	F^a	p	η_p^2
AR positive	0.717 (0.759)	1.101 (0.867)	1.288 (1.071)	1.369 (1.083)	4.707	.792	.051	0.186	> .999	.002	0.328	> .999	.004
AR negative	3.253 (1.143)	2.701 (1.430)	1.421 (1.043)	0.994 (1.084)	58.043	.030	.400	0.884	> .999	.010	0.065	> .999	.001
SFA	2.266 (0.871)	1.885 (0.917)	1.268 (0.826)	1.102 (0.767)	24.973	.030	.223	2.174	> .999	.024	1.725	> .999	.019
External focus	1.647 (0.931)	1.421 (1.047)	0.861 (0.771)	0.771 (0.757)	16.352	.030	.158	5.143	.650	.056	0.276	> .999	.003
PEP positive	0.739 (0.777)	1.107 (1.022)	1.253 (1.210)	1.496 (1.249)	3.442	> .999	.043	0.125	> .999	.002	0.818	> .999	.011
PEP negative	3.104 (1.295)	2.654 (1.442)	1.054 (0.984)	0.748 (1.008)	63.095	.030	.450	0.132	> .999	.002	0.63	> .999	.001

Note. SAD = Social anxiety disorder group. HC = Healthy control group. T1 = Session 1. T2 = Session 2. AR positive and AR negative measured by *TQ-C AR*[7,8], range: 0-5. SFA and External focus measured by *FAQ*[9,10], range: 0-4. PEP positive and PEP negative measured by *TQ-C PEP*[11,12], range: 0-5. Corrected *p*-values according to Bonferroni-Holm[2].

[a] $F(1,88)$ for AR positive, AR negative, SFA and External Focus. $F(1,78)$ for PEP positive and PEP negative (difference due to missing data).

Supplementary Table S2

Interaction Effects of ANOVAs with Age as a Covariate

Dependent variable	*M* (*SD*)				*F* tests					
	SAD		HC		Group x Session			Session x Age (in months)		
	T1	T2	T1	T2	F^a	p	η_p^2	F^a	p	η_p^2
AR positive	0.717 (0.759)	1.101 (0.867)	1.288 (1.071)	1.369 (1.083)	2.812	> .999	.031	0.017	> .999	.000
AR negative	3.253 (1.143)	2.701 (1.430)	1.421 (1.043)	0.994 (1.084)	0.142	> .999	.002	0.142	> .999	.002
SFA	2.266 (0.871)	1.885 (0.917)	1.268 (0.826)	1.102 (0.767)	4.350	.920	.048	0.793	> .999	.009
External focus	1.647 (0.931)	1.421 (1.047)	0.861 (0.771)	0.771 (0.757)	0.334	> .999	.004	6.535	.312	.070
PEP positive	0.739 (0.777)	1.107 (1.022)	1.253 (1.210)	1.496 (1.249)	0.304	> .999	.004	0.002	> .999	.000
PEP negative	3.104 (1.295)	2.654 (1.442)	1.054 (0.984)	0.748 (1.008)	0.490	> .999	.006	0.005	> .999	.000

Note. SAD = Social anxiety disorder group. HC = Healthy control group. T1 = Session 1. T2 = Session 2. AR positive and AR negative measured by *TQ-C AR*[7,8], range: 0-5. SFA and External focus measured by *FAQ*[9,10], range: 0-4. PEP positive and PEP negative measured by *TQ-C PEP*[11,12], range: 0-5. Corrected *p*-values according to Bonferroni-Holm[2].

[a] $F(1,88)$ for AR positive, AR negative, SFA and External Focus. $F(1,78)$ for PEP positive and PEP negative (difference due to missing data).

References

1. Vasey, M. W. & Thayer, J. F. The Continuing Problem of False Positives in Repeated Measures ANOVA in Psychophysiology: A Multivariate Solution. *Psychophysiology* **24**, 479–486 (1987).
2. Holm, S. A Simple Sequentially Rejective Multiple Test Procedure. *Scand J Statist* **6**, 65–70 (1979).
3. Faul, F., Erdfelder, E., Lang, A.-G. & Buchner, A. G*Power 3: A flexible statistical power analysis program for the social, behavioral, and biomedical sciences. *Behavior Research Methods* **39**, 175–191 (2007).
4. WHO World Mental Health Survey Collaborators *et al.* The cross-national epidemiology of social anxiety disorder: Data from the World Mental Health Survey Initiative. *BMC Med* **15**, 143 (2017).
5. Clark, D. M. & Wells, A. A cognitive model of social phobia. in *Social phobia: Diagnosis, assessment, and treatment* (eds. Heimberg, R. G., Liebowitz, M. R., Hope, D. A. & Schneier, F. R.) 69–93 (The Guilford Press, New York, 1995).
6. Kendall, P. C. & Chansky, T. E. Considering cognition in anxiety-disordered children. *Journal of Anxiety Disorders* **5**, 167–185 (1991).
7. Penney, E. S. & Abbott, M. J. The Impact of Perceived Standards on State Anxiety, Appraisal Processes, and Negative Pre- and Post-event Rumination in Social Anxiety Disorder. *Cogn Ther Res* **39**, 162–177 (2015).
8. Lidle, L. R. & Schmitz, J. Rumination in Children with Social Anxiety Disorder: Effects of Cognitive Distraction and Relation to Social Stress Processing. *Res Child Adolesc Psychopathol* **49**, 1447–1459 (2021).
9. Kley, H., Tuschen-Caffier, B. & Heinrichs, N. Safety behaviors, self-focused attention and negative thinking in children with social anxiety disorder, socially anxious and non-anxious children. *Journal of Behavior Therapy and Experimental Psychiatry* **43**, 548–555 (2012).
10. Kley, H., Tuschen-Caffier, B. & Heinrichs, N. Manipulating Self-Focused Attention in Children with Social Anxiety Disorder and in Socially Anxious and Non-Anxious Children. *Journal of Experimental Psychopathology* **2**, 551–570 (2011).
11. Schmitz, J., Krämer, M. & Tuschen-Caffier, B. Negative post-event processing and decreased self-appraisals of performance following social stress in childhood social anxiety: An experimental study. *Behaviour Research and Therapy* **49**, 789–795 (2011).
12. Schmitz, J., Krämer, M., Blechert, J. & Tuschen-Caffier, B. Post-event Processing in Children with Social Phobia. *J Abnorm Child Psychol* **38**, 911–919 (2010).
